# Odyssey of a small-town midwestern boy to a scholarly path

**DOI:** 10.1038/s41430-020-0577-8

**Published:** 2020-02-10

**Authors:** Barry M. Popkin

**Affiliations:** Department of Nutrition, Gillings School of Global Public Health, and the Carolina Population Center, The University of North Carolina at Chapel Hill, Chapel Hill, NC USA

**Keywords:** Nutrition, Health sciences

## My upbringing and education

I grew up in Superior, Wisconsin, in a strongly connected, education-oriented family. My father was a high school graduate, and my mother spent 1 month in college before leaving to support her siblings. Both of my parents, as well as my grandmother, stressed the importance of education. Every Sunday we visited my mother’s large clan in Duluth for a full day of play. I with eight or ten cousins would laugh and run, while my parents conversed with the other adults. We were close, literally. Within 100 m, I could find my family, my grandmother, who was next door as was my uncle and aunt, who lived with their two kids. Annual family gatherings were normal, and family was important, a strong element in my life.

My family encouraged good grades, a strong work ethic, and saving money. In my early childhood I shoveled snow, did garden chores, and mowed numerous large midwestern lawns for 8–12 families. I had a newspaper route in the evening, rain or shine. Funding during my age 12–17 year period came from running a baseball league and writing sports for a local newspaper. In high school, I did very well, even though I never studied outside of school. The school did not emphasize writing and this poor school did not teach me to study. Very few of my classmates continued to college (2–3%). My brother, the boy genius, began his brilliant career with a full scholarship at the Massachusetts Institute of Technology and wanted me to go to Ivy League. I opted for the honors program and a scholarship at the University of Wisconsin-Madison (UW), where I enrolled in 1962.

College was a shock. As a freshman I made money playing bridge and earned many masters points in regional tournaments. After my first semester of B+s, I decided it was not good enough. I dropped out of bridge and studied hard to right my ship. I primarily studied for exams and still had not learned to think. All that changed in 1965. In my senior year, I received a full scholarship to study Hindi for a month and travel to India with a group of students for the year. My life was not the same after that experience. During my year in India, I began to read books and have discussions on Vietnam with others who were part of this program. One of my friend’s father was close to Adlai Stevenson, and we attended his funeral. All these experiences plus the India experience opened me up intellectually.

I felt unprepared relative to my fellow students that went to India who were much more sophisticated and knowledgeable about the world than me, but by the end of that year I had changed. I studied economics and slum redevelopment, urban planning. When I arrived in India, I created a project looking at urban redevelopment of a huge *jhuggi jhompri*, an ultradense Indian squatter area. There were so many languages and dialects I did not know; I worked with an Indian interviewer who assisted me in interviewing people to understand their living conditions. My time in the *jhuggi jhompri* truly woke me to the world of poverty, exploitation, and the complexities of life. I lived there part-time and also spent time with my interviewer and his family in a village in the northern Punjab.

The United States bombed North Vietnam and Hanoi. Our student group protested with a demonstration that shut down the American Embassy. I was not a leader, but an active participant. The CIA interviewed us and tried to boot us out of India, while the Indian Government loved us and stopped it. My parents suffered due to local backlash as news reached my small town. The front page of *Time* magazine and *New York Times* articles, among others, highlighted this demonstration. Residents of my hometown burned torches and swastikas on my parents’ lawn.

As my time in India had come to an end, I used my Pan Am round-the-world ticket and $300 from a dear aunt to tour the great cities of Asia for 3 months. I traveled home on less than $5 a day, living like a king. I visited slums and other sites in Nepal, Thailand, Kuala Lumpur, and Singapore. I visited friends in the Philippines and traveled to Hong Kong. In Japan, I spent 3 weeks in a village near Gifu with the family of a student I had met in Singapore. This family spoke only Japanese, so it was an interesting experience. The last stop was Tokyo and then home. By the end, I was certain I wanted to be involved in international economic development work; moreover, this entire experience opened me to other perspectives and different people and it politicized me.

Back home in the United States, I did a senior thesis and selected the economics of nutrition. I wrote a small book on all its aspects, from the causes and consequences of undernutrition to the economic benefits of eliminating undernutrition. This included a focus on undernutrition in the United States. I obtained a Woodrow Wilson fellowship with a full ride and did my first year of economics graduate school at the Wharton School of Business at the University of Pennsylvania (UPenn). I took economics classes and conducted summer research with Richard Easterlin who had me take some demography courses and conducted my research work on measuring diets and nutritional status historically. However, I did not like UPenn’s economics training and the way Easterlin was studying historical trends. In August of 1969, I returned to UW where I excelled in graduate economics classes and got super involved in civil rights activities. I was a leader in a UW student strike that led to a shutdown and 600 national guardsmen on campus. After that year for the summer, the US Senate Hunger committee, led by George McGovern, gave me funding to report on the economics of undernutrition that led to a few publications and senate report [1, 2].

I left for several years of leftist organizing after my graduate studies without a dissertation. This was a period in which I worked with the Black Panthers and a cohort of organizers on an array of full-time activities. I left after a 2-year period. The main reason was a feeling of a lax of camaraderie and support among white activists such that I did not see us building a healthy future for our society.

I returned to UW to concentrate on a large poverty-related research project and then left for a job in Boston. In the summer of 1972, I met Jim Levinson, who focused on nutrition and got his PhD in agricultural economics from Cornell. He introduced me to Cornell faculty members, Michael Latham and David Call, who were both interested in nutrition issues. They offered me a research associate position at Cornell to establish their international nutrition program (September 1972) and funded my PhD program, which I began in January 1973. This was an opportunity I could not refuse and immediately said yes!

While at Cornell, I was invited to the 1972 International Conference on Nutrition in Mexico, where I met Dr. Florentino Solon, a nutrition visionary wedded to the reality of malnutrition, including micronutrient deficiencies, particularly vitamin A deficiency, in the rural Philippines. We designed a random controlled trial (RCT) in 12 communities with three arms and won grant funding. I spent the summer of 1973 in the Philippines designing the survey baseline. Jumping on a fast track, I collected the data that winter, wrote my dissertation, and graduated in the summer of 1974 after 18 months of doctoral study at Cornell [3]. My thesis and its data drew me into time-use research. I initiated a series of studies considering the time costs of breastfeeding, that is, breastfeeding versus market work, and the trade-offs poor women faced between work, infant care, and feeding [4]. This became an early line of work.

## My scholarly journey

### Research and surveys linking nutrition with economics

I decided to delve deeper into field research and lived abroad to develop a broader economics-oriented nutrition perspective. A job took me to Manila as a Rockefeller Foundation Field Staff member with a position at the prominent University of the Philippines School of Economics (UPSE), which was populated with economists trained at the University of Chicago, Harvard, and other notable American universities. The US Agency for International Development (USAID) provided funding for me to document the full-scale impact of the earlier vitamin A RCT.

I worked with amazing economists at the UPSE. I met Bob Evenson, a professor on leave from Yale University. He was working in the Agricultural Economics Department at the University of the Philippines Los Baños (UPLB) as a representative of the Agricultural Development Corporation led by Vern Ruttan. Bob and I started an intensive set of multipurpose surveys called the Laguna Village Surveys [5]. I insisted that we include time use, dietary intake, and body composition along with detailed income and demographic data. Our students used these data plus an additional in-depth study of a subset of 100 households in which we recorded 100% of the time use for each family member by sending an interviewer to each home for 3 days.

In addition, I led an extensive evaluation of a rural development project that included roads, electrification, clinics, and other health improvements with an economics aspect for USAID. I was the driving force behind the Bicol Multipurpose Survey and a follow-up survey across this region. Those data and several resurveys are now open to the public, and reside at Yale University Economic Growth Center.

In the Philippines, my students were economics graduates. We prepared many papers combining nutrition and economics, and these publications, along with my evaluation of the vitamin A RCT, added an economics perspective to the nutrition world. I also broadened my exploration of undernutrition, looking at the causes and effects, including several studies on breastfeeding. My work on demographics, economics, and nutrition pushed my interests into public health, and I wanted to return to a US university. I needed to be somewhere I could bike, which is a simple pleasure in my life. I had taken my bike to Manila, but riding there was too dangerous, as drivers ran me off the road. I declined positions at several urban institutions—positions where biking was not safe, and accepted a lucky offer from the University of North Carolina at Chapel Hill (UNC) Department of Nutrition.

At UNC, I talked to two outstanding economists (John Akin and David Guilkey) into joining my nutrition and public health research world. We received funding to evaluate the US national school lunch and breakfast programs by using nationally representative surveys. Later, we undertook studies across the globe on breastfeeding patterns and determinants [6]. This was during the Nestle boycott era. A major gift allowed us to organize the “Rolls Royce” of longitudinal surveys, the Cebu Longitudinal Health and Nutrition Survey [7], that followed women from pregnancy through birth (within 3 days of birth) with follow-ups every 2 months for 2 years. These individual, household, and community surveys allowed sophisticated longitudinal dynamic models of breastfeeding and total infant-feeding patterns, determinants, and consequences for growth, diarrheal and respiratory status, and deaths of infants, and had an impact on mothers and maternal nutrition. I obtained my first three NIH R01’s for that research.

### The origins of my nutrition transition thinking

In the Philippines, I had realized that societal changes—inflation, weather, and other external forces not determined by individual or household behaviors—greatly impact health and nutrition. I decided to transition my Cebu research to a colleague, Dr. Linda Adair, and develop a nationwide survey. My investigations determined the value and importance of conducting this survey in China, which was just beginning national reforms. It took us 3 years to find the right collaboration. With the mentorship of a China sociologist, Gail Henderson, and an eminent Chinese economist, I concluded that the focus should be collaborating with a health institution. I met Chen Chumming, president of the Chinese Academy of Preventive Medicine, at an international meeting in 1976, and capitalized on a World Bank review of her institution that encouraged it to expand its social science focus and build analytic capacity. At UNC we offered both.

Madame Chen and I worked out a basis for collaboration, and in 1977 she visited UNC for 2 weeks meeting the chairs and key faculty in our Gillings School of Global Public Health. During her visit we conceptualized the China Health and Nutrition Survey [8]. We designed the survey, arranged a small grant through the Carolina Population Center, and conducted a shoestring-funded survey in 1989. Having demonstrated that we could conduct such a wide-ranging survey and could bring the data out of China, the first time in modern China’s history, we secured funding from the National Institutes of Health that is still ongoing.

Looking at change between the first few surveys, I discovered nutritional change that was both positive and negative. We saw more hunger and undernutrition increasing in selected poor countries, while we saw many other countries where adults were moving toward overweight/obesity. Considering these changes, I decided I needed to take time off to truly focus on the dynamic nutritional changes from a historical perspective. I read extensively on food and health history from the perspectives of anthropology, ethnography, evolution, and agriculture. To solidify what I discovered, I wrote an unpublished treatise of several hundred pages. This led to my theory of the nutrition transition and my understanding of the clash between our evolutionary biology and modern technology in how we eat, drink, and move.

I published case studies, wrote several more conceptual papers, and established a way of looking at the dynamics of food-eating behavior, drinking transitions, the biology of hydration, time-use changes, and labor-saving technology. These changes greatly impact our ways of eating, drinking, and moving and subsequently our body composition and health. Also, during that period, a doctoral student and I conducted the first study on the dual burden of malnutrition.

### Deepening my understanding of obesity

It took me decades to convince my colleagues in nutrition and public health of the coming global obesity epidemic and I cemented that with a Bellagio meeting I organized in 2002 [9]. As recognition of the nutrition transition became accepted, I turned to the task of explaining to the field the need to create large-scale regulatory and fiscal actions at the national level to prevent the changes linked with the current transition stage of nutrition-related noncommunicable diseases and the behaviors that negatively affect all aspects of nutrition and health. At a second Bellagio Conference, colleagues adopted the mantra and pushed for change, leading me to the final phase of my odyssey—creating a literature on policies that work and do not work with a tool package of defensible high-impact actions at the national level [10].

My research career has always focused on studying social, dietary, physical activity, and body composition changes and their dynamics. I see a major need for more scholars in all public health fields to consider these dynamics and not stop with a focus on the short-term and cross-sectional surveys. I see a need to understand how forces exogenous to the household are creating and changing how we eat, drink, and move. I see a need for others to study options for creating healthy lifestyles at the household, community, and national levels. We need countries to adopt the most impactful policies linked to removing ultra-processed food from our diet and shifting toward truly healthful eating and eventually healthy lifestyles. This will ultimately require a shift in eating norms and the creation of an environment focused on healthy eating.

One activity I undertook was writing a very easy to digest book “The World is Fat” which was published in 13 languages to explain these changes [11]. For this last phase of my active research and research-related advocacy, I am focused on creating national policies, regulations, and laws focused on healthy eating, including consulting with countries and multinational agencies on this agenda, and continued scholarship in this area.
